# Association between Beverage Consumption and Environmental Sustainability in an Adult Population with Metabolic Syndrome

**DOI:** 10.3390/nu16050730

**Published:** 2024-03-03

**Authors:** Silvia García, Margalida Monserrat-Mesquida, Emma Argelich, Lucía Ugarriza, Jordi Salas-Salvadó, Inmaculada Bautista, Jesús Vioque, María Dolores Zomeño, Dolores Corella, Xavier Pintó, Aurora Bueno-Cavanillas, Lidia Daimiel, J. Alfredo Martínez, Stephanie Nishi, Estefanía Herrera-Ramos, Sandra González-Palacios, Montserrat Fitó, Eva M. Asensio, Marta Fanlo-Maresma, Naomi Cano-Ibáñez, Esther Cuadrado-Soto, Itziar Abete, Josep A. Tur, Cristina Bouzas

**Affiliations:** 1 CIBER Fisiopatología de la Obesidad y Nutrición (CIBEROBN), Instituto de Salud Carlos III (ISCIII), 28029 Madrid, Spain; silvia.garcia@uib.es (S.G.); margalida.monserrat@uib.es (M.M.-M.); eargelich15@gmail.com (E.A.); jordi.salas@urv.cat (J.S.-S.); mfito@imim.es (M.F.); iabetego@unav.es (I.A.);; 2 Research Group on Community Nutrition & Oxidative Stress, University of the Balearic Islands—IUNICS, 07122 Palma de Mallorca, Spain; 3 Health Research Institute of the Balearic Islands (IdISBa), 07120 Palma de Mallorca, Spain; 4 Departament de Bioquímica i Biotecnologia, Unitat de Nutrició, Universitat Rovira i Virgili, 43201 Reus, Spain; 5 Institut d’Investigació Sanitària Pere Virgili (IISPV), 43201 Reus, Spain; 6 Research Institute of Biomedical and Health Sciences (IUIBS), University of Las Palmas de Gran Canaria & Centro Hospitalario Universitario Insular Materno Infantil (CHUIMI), Canarian Health Service, 35016 Las Palmas de Gran Canaria, Spain; faniya1@gmail.com; 7 Instituto de Investigación Sanitaria y Biomédica de Alicante, Universidad Miguel Hernández (ISABIAL-UMH), 03550 Alicante, Spain; vioque@umh.es (J.V.);; 8 CIBER de Epidemiología y Salud Pública (CIBERESP), Instituto de Salud Carlos III, 28029 Madrid, Spain; abueno@ugr.es (A.B.-C.);; 9 Unit of Cardiovascular Risk and Nutrition, Institut Hospital del Mar de Investigaciones Médicas Municipal d`Investigació Médica (IMIM), 08003 Barcelona, Spain; 10 School of Health Sciences, Blanquerna-Ramon Llull University, 08025 Barcelona, Spain; 11 Department of Preventive Medicine, University of Valencia, 46100 Valencia, Spaineva.m.asensio@uv.es (E.M.A.); 12 Lipids and Vascular Risk Unit, Internal Medicine, Hospital Universitario de Bellvitge—IDIBELL, Hospitalet de Llobregat, 08907 Barcelona, Spain; 13 Department of Preventive Medicine and Public Health, University of Granada, 18016 Granada, Spain; 14 Nutritional Control of the Epigenome Group, Precision Nutrition and Obesity Program, IMDEA Food, CEI UAM + CSIC, 28049 Madrid, Spain; 15 Departamento de Ciencias Farmacéuticas y de la Salud, Facultad de Farmacia, Universidad San Pablo—CEU, CEU Universities, Urbanización Montepríncipe, 28660 Boadilla del Monte, Spain; 16 Precision Nutrition and Cardiometabolic Health Program, IMDEA Food, CEI UAM + CSIC, 28049 Madrid, Spain; 17 Department of Nutrition, Food Sciences, and Physiology, Center for Nutrition Research, University of Navarra, 31008 Pamplona, Spain; 18 VALORNUT Research Group, Department of Nutrition and Food Science, Faculty of Pharmacy, Complutense University of Madrid, 28040 Madrid, Spain

**Keywords:** beverages, drinks, sustainability, sustainability score, environmental parameters, health, metabolic syndrome

## Abstract

Beverages are an important part of the diet, but their environmental impact has been scarcely assessed. The aim of this study was to assess how changes in beverage consumption over a one-year period can impact the environmental sustainability of the diet. This is a one-year longitudinal study of 55–75-year-old participants with metabolic syndrome (n = 1122) within the frame of the PREDIMED-Plus study. Food and beverage intake were assessed using a validated food frequency questionnaire and a validated beverage-specific questionnaire. The Agribalyse^®^ 3.0.1 database was used to calculate environmental impact parameters such as greenhouse gas emission, energy, water, and land use. A sustainability beverage score was created by considering the evaluated environmental markers. A higher beverage sustainability score was obtained when decreasing the consumption of bottled water, natural and packed fruit juice, milk, and drinkable dairy, soups and broths, sorbets and jellies, soft drinks, tea without sugar, beer (with and without alcohol), and wine, as well as when increasing the consumption of tap water and coffee with milk and without sugar. Beverage consumption should be considered when assessing the environmental impact of a diet. Trial registration: ISRCTN, ISRCTN89898870. Registered 5 September 2013.

## 1. Introduction

The consumption of beverages other than water is significant on a global scale. The health effects of these beverages have already been studied [1], but there is still a knowledge gap concerning their environmental impact. Recently, an intervention study in children aimed to assess the possible interaction between beverages and sustainability, but the conclusions were unclear, claiming that more interventions based on nutrition and on environmental sustainability are needed [2]. The environmental impact can vary depending on the type of beverage. It has been observed that reducing the consumption of ultra-processed beverages could potentially decrease both the environmental footprint and overall mortality risk [3]. However, it is not entirely clear yet whether its consumption, production, packaging type, or other factors make each beverage more or less sustainable [4,5].

Beverage consumption is an important factor contributing to the population’s total intake as well. Specific drinks were studied due to their direct effects on health. An excessive consumption of sugar-sweetened beverages was found to be a risk factor for obesity, cardiovascular disease, type 2 diabetes, and dental caries, and several interventions were applied in different countries to decrease it [6]. Other relevant beverages with a negative impact on health were alcoholic drinks since their excessive consumption may lead to physical and mental disorders [7]. Recommendations on light and moderate alcohol consumption were presented to decrease mortality risk [7,8]. Usual beverages such as dairy products or coffee were also studied in relation to health. The increasing presence of chronic inflammatory diseases led to a study of their relationship with dairy product consumption since dairy can modulate human inflammatory processes [9]. Long-term coffee consumption effects were related with cardiovascular diseases [10]. The risk of metabolic syndrome (MetS) is also affected negatively or positively by beverage consumption [11,12]; however, MetS is closely related to lifestyle, and several factors could modify its severity [13].

The increasing presence of unhealthy and unsustainable foods and beverages leads the planet and the people to a global risk. Increases in non-communicable diseases together with the effects of food on greenhouse gas emissions (GHGs), fertilizer pollution, loss of biodiversity, energy use, water use, and land use will lead the Earth to a detrimental state. A more sustainable way of eating is necessary to meet the United Nations’ Sustainable Development Goals and other environmental protective strategies [14]. In 2015, 195 nations agreed on creating the Agenda 2030, which was aimed to accomplish 17 Sustainable Development Goals by the year 2030, making our world more environmentally friendly. The 17 goals are interconnected and some of them are related to food production, consumption, and security [15]. The food industry observed sustainable changes made in consumer purchases, and many producers have integrated environmental, social, or economic sustainability aspects into stages of their production processes [16].

Although it is known that beverages are an important part of diet composition and have different effects on health. The specific environmental impact of beverage consumption has been scarcely analyzed. The aim of this study was to assess how changes in beverage consumption over a one-year period can impact the environmental sustainability of the diet.

## 2. Methods

### 2.1. Study Design

The current study is a one-year longitudinal study within the frame of the PREDIMED-Plus trial. It is a parallel-group, randomized, and controlled trial, which aimed to combine an energy-reduced traditional Mediterranean Diet (MedDiet) with physical activity, and behavioral support to see its effects on cardiovascular disease morbimortality. The study protocol can be found elsewhere [17] and at https://www.predimedplus.com/en/, accessed on 5 September 2013. The International Standard Randomized Controlled Trial (ISRCT; http://www.isrctn.com/ISRCTN89898870, accessed on 5 September 2013) registered the mentioned trial with the number 89898870 in 2014.

### 2.2. Participants, Recruitment, Randomization, and Ethics

**Figure 1:**
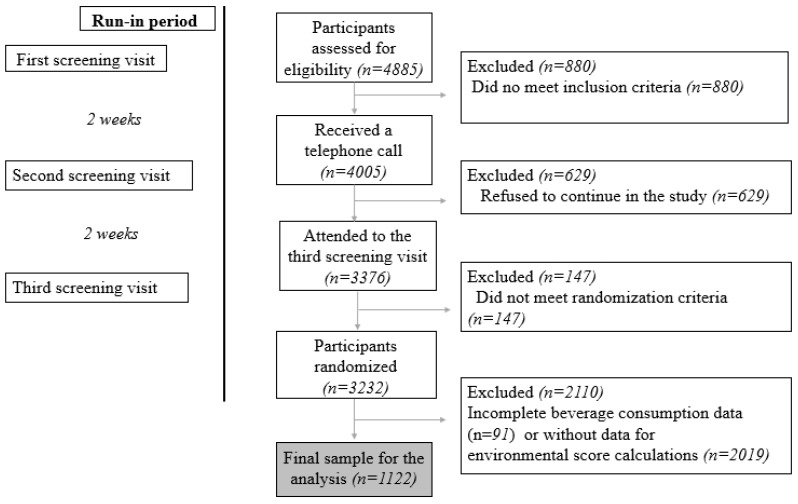
Flow chart of eligibility of participants.

The PREDIMED-Plus beverage group was composed of 3232 participants aged 55–75 years old with a body mass index (BMI) of 27–40 kg/m^2^, meeting three or more criteria of the MetS according to the International Diabetes Federation and the American Heart Association/National Heart, Lung, and Blood Institute [18], and with available beverage consumption data. Participants who did not fully complete the beverage consumption questionnaire were excluded (n = 2110), as their environmental impact could not be accurately calculated. The final sample was 1122 participants. A flow-chart of eligible participants is shown in Figure 1. All participants were provided with an informed written consent. The study protocol and procedures were approved by Ethics Committee of Research of Balearic Islands (refs. CEIC-IB2251/14PI and CEIC-IB1295/09PI; approved on 26 February 2020, and 2 July 2022, respectively), following the ethical standards of the Declaration of Helsinki.

### 2.3. Assessment of Food and Beverage Intake

Food and beverage intake were assessed separately by trained dietitians with questionnaires at baseline and after one-year follow-up. A semi-quantitative 143-item food frequency questionnaire (FFQ) previously validated in a Spanish population [19,20] was used to record food intake. Consumption frequencies were registered according to 9 categories (from “never or almost never” to “≥6 times/day”) and a regular portion size was established for each item. A computer program based on Spanish food composition information was used to calculate energy and nutrient intakes [21,22]. Results were used to assess the total energy intake consumed (kcal) for each participant per day. Beverage intake assessment was performed with a previously validated beverage-specific questionnaire [23]. The daily and weekly beverage consumption of 32 beverages consumed during the previous month was recorded and estimations on the average daily beverage intake were performed based on servings of each type of beverage. The sum of all different beverages was considered as the total beverage intake. Table 1 shows the questionnaire items of beverages.

### 2.4. GHGs, Energy, Water, and Land Use per kg of Food

Environmental parameter calculations were conducted using the Agribalyse^®^ 3.0.1 database created by the French Agency for the Environment and Energy Management (ADEME), in conjunction with CIQUAL French food composition table [24]. Ecoinvent^®^ also cooperates with Agribalyse^®^ 3.0.1; data are stored in the Ecoinvent database for non-agricultural procedures (e.g., electricity, transport) and imported production; together, they aim to reflect the production and market conditions of European countries. The Agribalyse^®^ 3.0.1 database provides reference data on the environmental impacts of agricultural and food products through a database built according to the LCI methodology [25]. It considers each phase in the food chain separated in two steps: production and post-farm procedures. Agricultural production, transport, processing, packaging, distribution and retailing, consumer preparation, and disposal of packaging are the steps considered to measure environmental impacts. Wastage at home and transport from retail to the household is not included. The method is based on the international LCA standards: ISO 14040 [26] and ISO 14044 [27], LEAP guidelines [28], and product environmental footprint (PEF) [29]; final measurements of each environmental indicator are provided per kg of product. Total amounts of GHGs, water use, energy use, and land use were used for the present paper and are described below.

GHGs were expressed in kilograms (kg) of carbon dioxide equivalents (CO_2_eq). Water use was calculated in cubic meters (m^3^) and in correspondence with water consumption and depletion in certain regions, taking scarcity into account. Energy use was calculated in megajoules (MJ) and corresponded to the disposal of non-renewable energy resources like carbon, gas, oil, or uranium. Land use largely determines biodiversity. The unit used for the variable land use was estimated using the echo indicator point (Pt), which reflects the impact of an activity on land biodiversity degradation with reference to the “natural state”, meaning that higher levels of land degradation would be reflected with higher Pt units.

All four parameters were estimated at baseline and 1-year follow-up using the following formula:

$$=\frac{g\;\mathrm{of}\;\mathrm{each}\;\mathrm{reported}\;\mathrm{food}\times \mathrm{Amount}\;\mathrm{of}\;\mathrm{the}\;\mathrm{Environmental}\;\mathrm{Parameter}}{1000\;g\;\mathrm{of}\;\mathrm{the}\;\mathrm{corresponding}\;\mathrm{food}}$$

The sum of the total diet’s impact was calculated for each parameter separately. Differences between baseline results and one-year results per day was also calculated.

### 2.5. Environmental Score Calculations

A sustainability score for beverages was calculated considering all four environmental parameters: GHGs (kgCO_2_eq), energy use (MJ), water use (m^3^), and land use (Pt). The environmental impact of beverage consumption was calculated for each of these environmental parameters, as explained in the previous section. Subsequently, the medians of each environmental parameter were calculated and used as cut-off points. Values above medians were scored as 0, and values below medians were scored as 1. When scores of the four environmental parameters were added together, we ended up with a range from 0 to 4, with higher scores indicating lower environmental impact [30]. Score differences between baseline and one-year results were also calculated.

### 2.6. Sociodemographic Characteristics

Sociodemographic characteristics such as sex, age, and scholar level were also obtained.

### 2.7. Statistical Analyses

SPSS statistical software package version 27.0 (SPPS Inc., Chicago, IL, USA) was used to perform the analyses. Medians of CO_2_eq emissions, m^3^ of water, MJ of energy, and Pt of land for beverage consumption were calculated in order to create the beverage sustainability score. Data on beverage sustainability score change per participant and day between baseline and the first year were distributed in tertiles: tertile 1 (T1), participants with a reduction in their score (−4 to −1 points); tertile 2 (T2), participants with no changes in their score (0 points); and tertile 3 (T3), participants with an increase in their score (1 to 4 points). Data were shown as mean and standard deviation (SD), except for prevalence data, which were expressed as sample size and percentage. Chi-squared test was used for categorical variables and one-way ANOVA and Bonferroni’s post hoc test was used for continuous variables. General linear model (GLM) was used to relate changes in the beverage sustainability score and the beverages consumed by participants during one-year follow-up. GLM was adjusted by total energy intake and intervention group.

## 3. Results

Table 2 shows sociodemographic characteristics of the sample at baseline. Sex, educational level, age, and BMI were homogeneously distributed among the three categories of beverage score sustainability.

**Figure 2:**
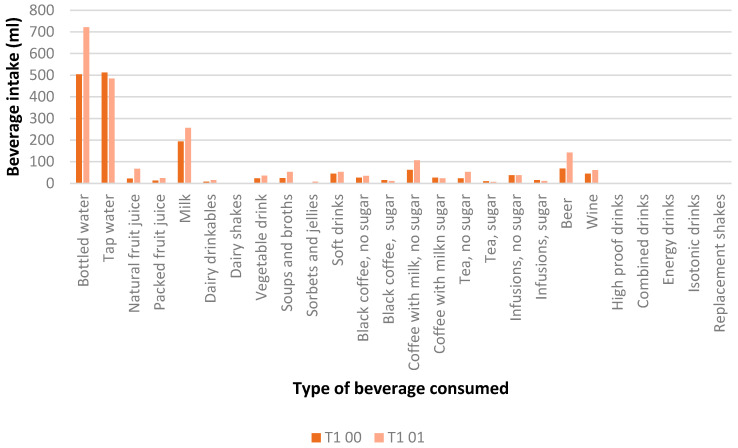
Differences between beverage intake (mL) at baseline (00) after one-year follow-up (01) in the low sustainable group (T1).

**Figure 3:**
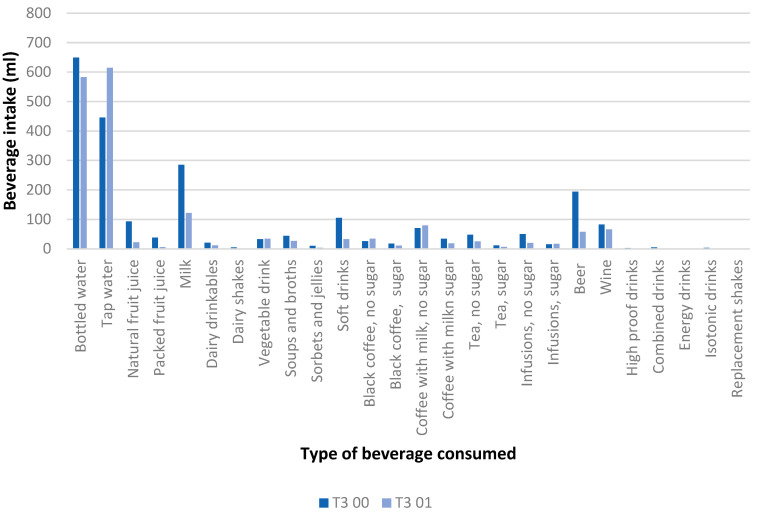
Differences between beverage intake (mL) at baseline (00) and after one-year follow-up (01) in the high sustainable group (T3).

Table 3 shows changes in beverage sustainability score after one-year follow-up, showed by beverage consumed. The group with a higher beverage sustainability score (tertile 3: T3) showed decreased consumption of bottled water, natural and packed fruit juice, milk, and drinkable dairy, soups and broths, sorbets and jellies, soft drinks, tea without sugar, beer (with and without alcohol), and wine, as well as an increased consumption of tap water and coffee with milk and without sugar. The group with a lower beverage sustainability score (tertile 1: T1) consumed more bottled water, but not coffee with milk and without sugar. These differences in beverage consumption between the low sustainable group (T1) and the high sustainable group (T3) during the one-year period, are shown in Figure 2 and Figure 3.

## 4. Discussion

Beverage consumption was related with GHGs, water use, energy use, and land use. Some beverages appeared to be more environmentally friendly than others, reflecting how beverage consumption should be considered when assessing the environmental impact of a diet.

Given that each beverage has a distinct life cycle, previous studies assessed its environmental impact individually. The sustainability of the milk and dairy industry was addressed from 2011 since it was found to be among the most pollutant industries [31]. The extensive production of milk and its derivatives results in high resource consumption and a significant volume of waste, with whey production being particularly problematic [32]. Among environmental indicators, water use was found as the one that stands out above all [33]. Milk and dairy products are usually consumed, due to their valuable nutrient composition. This is why sustainable practices should be implemented in the dairy industry, considering environmental, economic, and social sustainability [34,35,36]. Current results show that milk consumption decreased more than half in the population group with the highest sustainability score, while its reduction is not as high in the other population groups.

The environmental pressure of the food system would be attenuated by moving towards a more plant-based (PB) diet [37,38]. Demands of PB products increased over the last several years [38,39]. However, not all of the population is willing to change their food habits; a lack of environmental consciousness or a phobia of new food products were seen in study results [40,41]. Current results show no relationships between the beverage sustainability score and vegetable drink consumption, perhaps due to the low consumption of these products in our studied population. Low vegetable drink consumption among current participants could be understood due to their age; they are older than 55 years old. The rejection of new foods is usual in children, but also in older people, mainly due to established traditional and cultural habits [42]. Age implies inherent differences in health status, behaviors, and life experiences between older and younger individuals. Factors such as physiological changes, generational disparities in technology use, and evolving social norms could affect the outcomes of the current study in younger age groups [43,44,45].

Alcoholic beverages, particularly wine, are part of the MedDiet. Wine and beer are the most consumed alcoholic beverages among our study population. Reductions were observed in the consumption of both, with a notable decrease seen in beer consumption. Although both reductions were statistically significant, the decrease in wine consumption was minimal. This can be explained because in the current study population, the participants were recommended to follow the MedDiet. It has been seen how red wine, consumed in moderation, can reduce the risk of MetS [46] and also contribute to improving cardiovascular health [47]. That is the reason why one of the 17 items to assess the MedDiet adherence is related to wine consumption, and it is scored positively if consumption was one drink/day for women and two drinks/day for men [48]. From an environmental perspective, wine and beer production were the most impactful sectors, especially because of water use, energy use, and wastewater [49,50]. Several studies calculated GHGs emitted from wine and beer industries and found out that a decrease in its consumption will decrease GHGs [49,50,51]. The present study shows more holistic results, since it considers GHGs, water use, energy use, and land use to calculate the environmental score. It seems that rather than focusing on reducing its consumption, proposals such as decreasing the use of fertilizers and pesticides [34] or using renewable energies [50] can help to achieve a sustainable production of these beverages. Most of the previous studies focused on the management and reuse of derivative and waste products [52,53]. The reuse of lees from wine, beer, and cider was possible by converting them in yeast extract, nutritional supplements, or fertilizers; by performing a recovery of ethanol via distillation; by producing biogas; or by using it for animal feeding [52]. Yeasts used for wine fermentation are a reusable source for developing new strains or making mixed yeast mixtures to achieve more efficient wine fermentation [53]. Another challenge would be to convince people to buy sustainable beverages. In the case of sustainable beer, its composition or effects on human health would not change, but it is more expensive [54]. However, it was found that the US population would be willing to pay more for sustainably brewed beer, mostly among those conscious of their consumption impact [54].

Coffee and tea are also two of the most consumed non-alcoholic beverages worldwide, and their production and consumption are increasing, as well as their environmental impact [55,56]. Current results for hot beverages were inconclusive. Most of the hot beverages studied did not exhibit statistical significance, except for coffee with milk without sugar and tea without sugar, which were significative but showing low size effect differences. It appears that the consumption of these kinds of beverages did not impact sustainability as much as their production methods. This is the reason why a set of sustainability standards for the agriculture of these products was assessed a few years ago [57]. The sustainability of coffee and tea is based in improving agricultural techniques [58,59], such as improving pesticide usage [60] or reusing waste products [55,56]. Spent coffee grounds have a great composition to be reused as biopolymers, biofuels, activated carbon, filler material, and fertilizers, as well as converted into pharmaceuticals, materials, and energy production [55]. Tea waste products can be transformed into absorbents such as waste biochar and activated carbon, which could absorb pollutant compounds from water, air, and soil and would be cheaper than its commercial equivalents [56]. Comparing black, oolong, and green tea, green tea appeared to be the more sustainable one in terms of energy use, being also the healthiest because of its great mineral value [61].

Sugar-sweetened beverages are identified as ultra-processed products, which are related with higher GHGs and pollution [11,62]. One study found that college students who took a footprint seminar reduced their environmental impact, reporting a reduction in their sugar-sweetened beverage intake [63]. Soft drinks were reduced by more than half in the population group with the highest sustainability score but increased in the group with the lowest sustainability score. A replacement of these soft drinks with tap water would mean a reduction in GHGs [50]. Current results also showed that switching from bottled water to tap water would result in a better beverage sustainability score. Preference for bottled water was found to be higher because of its organoleptic characteristics and due to health concerns [64,65]. The sustainability of tap water is also reflected in other studies, and it may be more consumed if environmentally friendly water consumption was better known [66,67].

### Strengths and Limitations of the Study

The current paper offers a new source of information of a growing important issue: beverage sustainability. This paper has the following strengths. The literature reviewed assessed beverage sustainability separately, whereas in our study, the assessment considered all beverage consumption together, allowing calculations for the whole impact of the beverage intake. The analysis of environmental parameters was conducted by creating a holistic sustainable score, which includes GHGs, energy use, water use, and land use. The Agribalyse^®^ 3.0.1 database, recently updated in 2023, was consulted for environmental calculations by considering all the processing steps of a product life cycle. The PREDIMED-Plus study allows for the analysis of a large-sized sample. Using two validated questionnaires such as FFQ and a fluid-specific questionnaire makes the food and beverage intake record more reliable. Finally, the longitudinal design of the study allows for a causal evaluation.

Some limitations were also present in this study. Given that our participants were elderly, it is important to acknowledge that the findings and conclusions of our research may not directly apply to a younger population and cannot be readily generalized to support conceptual sustainability. Therefore, it is essential for future research to consider these age-related variations when attempting to extend our findings to a broader and more diverse demographic. The fact that the literature showed analyses of beverages separately made it more difficult to perform accurate comparisons with the findings of the current study. Comparisons are also limited because there are no references or validated models to calculate environmental scores. There is no reference database for environmental parameter calculations either. The environmental calculations in this study are based on data from the Agribalyse^®^ 3.0.1 database, which may have limitations in terms of the database’s comprehensive coverage of all beverages considered. In the current analysis, calculations for the beverage sustainability score encompassed all beverages showed in the study, except for tap water. In future studies on beverage sustainability, it is important to consider the use of pesticides and fertilizers, along with waste management. These are significant aspects frequently discussed in beverage production.

## 5. Conclusions

A higher beverage sustainability was observed in relation to a decreasing consumption of bottled water, natural and packed fruit juice, milk and drinkable dairy, soups and broths, sorbets and jellies, soft drinks, tea without sugar, beer (with and without alcohol), and wine, as well as an increasing consumption of tap water and coffee with milk and without sugar. Beverage consumption should be considered when assessing the environmental impact of a diet.

## Figures and Tables

**Table 1:** Fluid-specific questionnaire items.

- Tap water, bottled water, natural fruit juices, bottled fruit juices, natural vegetable juices, bottled vegetable juices. - Whole milk, semi-skimmed milk, skimmed milk, drinking yogurt (100–200 cc), milkshakes. - Vegetable drinks, soups. - Jellies and sorbets, sugar-sweetened beverages (SSBs) (200–330 cc), artificially sweetened beverages (ASBs) (200–330 cc). - Espresso (sweetened and unsweetened), white coffee (sweetened and unsweetened), tea (sweetened and unsweetened), other infusions (sweetened and unsweetened). - Beer (200–330 cc), non-alcoholic beer (200–330 cc), wine, spirits, mixed alcoholic drinks, energy drinks, sports drinks (200–330 cc), meal replacement shakes and other beverages.

**Table 2:** Sociodemographic characteristics at baseline.

	T1: Decreased Beverage Score (n = 374)	T2: Non-Changed Beverage Score (n = 360)	T3: Increased Beverage Score (n = 388)	*p*
Sex				0.308
Men (n; %)	173 (46.3)	178 (49.4)	201 (51.8)	
Women (n; %)	201 (53.7)	182 (50.6)	187 (48.2)	
Highest school level completed				0.696
Primary School (n; %)	45 (12.0)	36 (10.0)	50 (12.9)	
College School Technician (n; %)	30 (8.0)	31 (8.6)	36 (9.3)	
Secondary School (n; %)	97 (25.9)	91 (25.3)	109 (28.1)	
Bachelor’s degree (n; %)	202 (54.0)	202 (56.1)	193 (49.7)	
Age (years) *	64.9 (4.9)	65.0 (4.7)	64.8 (5.0)	0.832
BMI (kg/m^2^) *	32.6 (3.4)	32.6 (3.2)	33.1 (3.7)	0.188
Energy (Kcal/day) *	2400.3 (592.3)	2479.7 (618.7)	2531.7 (578.1)	0.009
Physical Activity (METs) ^	2297.5 (2256.2)	2611.1 (2301.9)	2478.1 (2484.2)	0.193

* Mean values (SD). Abbreviations: SD: Standard deviation. BMI: Body mass index. METs: Metabolic equivalents of task. Chi-squared test was used for categorical variables and one-way ANOVA and Bonferroni’s post hoc test was used for continuous variables. ^ Measured in METs (metabolic equivalents of task) min/week.

**Table 3:** Changes in beverage sustainability score after one-year follow-up showed by beverage consumed.

	T1: Decreased Beverage Score ^§^ (n = 374)	T2: Non-Changed Beverage Score ^§^ (n = 360)	T3: Increased Beverage Score ^§^ (n = 388)	*p*-Value Time × Group Interaction
Bottled water				<0.001
v00	503.8 (559.7) b	605.8 (593.9)	649.4 (645.2) b	
v01	721.7 (633.2) b	665.7 (654.8)	582.6 (635.9) b	
▲	217.8 (651.4) * d e	59.8 (539.1) * d f	−66.7 (669.1) * e f	
Tap water				<0.001
v00	511.8 (583.2)	453.8 (596.2)	445.9 (614.6)	
v01	484.8 (588.0) b	495.7 (619.9)	614.4 (681.1) b	
▲	−27.0 (588.4) e	41.9 (532.9) f	168.5 (651.8) * e f	
Natural fruit juice				<0.001
v00	22.5 (74.2) a b	83.4 (150.6) a	92.9 (157.3) b	
v01	67.9 (143.8) b	68.4 (148.9) c	22.4 (79.3) b c	
▲	45.3 (140.3) * d e	−15.0 (147.1) d f	−70.5 (161.9) * e f	
Packed fruit juice				<0.001
v00	12.9 (57.1) a b	34.7 (101.2) a	38.2 (110.8) b	
v01	23.8 (102.5) b	16.6 (79.5)	5.6 (33.2) b	
▲	10.8 (99.4) d e	−18.0 (110.8) * d	−32.5 (110.6) * e	
Milk (whole/semi-skimmed/skimmed)				<0.001
v00	193.9 (203.7) a b	242.2 (212.2) a c	285.3 (205.8) b c	
v01	256.3 (215.4) b	217.4 (230.3) c	121.4 (182.6) b c	
▲	62.3 (229.5) * d e	−24.8 (206.3) * d f	−163.9 (230.8) * e f	
Drinkable dairy				0.001
v00	8.2 (44.4) b	14.4 (57.8) b	20.5 (69.6)	
v01	14.8 (57.4)	8.3 (43.3)	11.8 (45.6)	
▲	6.6 (59.1) * d e	−6.1 (60.1) d	−8.7 (70.8) * e	
Dairy shakes				0.176
v00	0.4 (4.4)	3.4 (31.1)	4.9 (40.9)	
v01	2.9 (30.9)	2.2 (29.7)	1.3 (21.0)	
▲	2.5 (31.3)	−1.2 (31.2)	−3.5 (46.2)	
Vegetable drinks				0.257
v00	23.1 (90.5)	25.2 (99.9)	32.8 (112.7)	
v01	35.7 (116.4)	25.8 (96.5)	33.9 (108.6)	
▲	12.5 (111.1) *	0.6 (95.8)	1.1 (115.5)	
Soups and broths				<0.001
v00	24.2 (29.1) b	36.1 (70.9)	44.3 (80.6) b	
v01	53.4 (79.8) a b	39.8 (73.0) a c	26.6 (34.4) b c	
▲	29.2 (82.6) * d e	3.7 (91.5) d f	−17.7 (76.6) * e f	
Sorbets and jellies				0.002
v00	2.6 (13.4) b	6.3 (43.2)	10.4 (55.2) b	
v01	7.8 (40.6)	4.3 (31.5)	3.3 (23.9)	
▲	5.1 (38.6) * d e	−2.0 (32.0) d	−7.1 (57.7) * e	
Soft drinks (with/without sugar)				<0.001
v00	44.8 (113.1) a b	93.4 (230.3) a	105.1 (207.2) b	
v01	53.2 (148.3)	51.7 (135.1)	32.5 (103.3)	
▲	8.4 (168.1) d e	−41.6 (221.4) * d	−72.6 (204.3) * e	
Black coffee without sugar				0.852
v00	25.9 (38.4)	21.6 (37.6)	25.9 (44.6)	
v01	34.4 (43.5)	31.1 (43.8)	34.2 (42.5)	
▲	8.5 (46.1) *	9.4 (46.8) *	8.2 (54.0) *	
Black coffee with sugar				0.477
v00	14.8 (32.8)	17.5 (34.1)	17.7 (35.2)	
v01	11.0 (1507)	14.1 (32.3)	10.9 (27.7)	
▲	−3.8 (34.7) *	−3.4 (37.2)	−6.8 (38.3) *	
Coffee with milk without sugar				0.008
v00	62.6 (113.3)	57.8 (104.7)	70.3 (116.7)	
v01	106.4 (124.7) b	91.2 (121.4)	79.3 (116.7) b	
▲	43.8 (147.2) * e	33.3 (127.9) *	9.0 (148.3) e	
Coffee with milk with sugar				0.488
v00	26.8 (83.2)	35.9 (89.5)	34.4 (85.2)	
v01	22.8 (74.5)	26.8 (76.1)	18.1 (63.9)	
▲	−1.0 (100.6)	−9.1 (101.1)	−16.3 (101.5) *	
Tea without sugar				<0.001
v00	23.3 (90.4) b	35.8 (104.6)	48.1 (125.8) b	
v01	52.8 (156.7) b	37.6 (106.3)	25.2 (82.7) b	
▲	29.4 (155.3) * d e	1.8 (125.8) d f	−22.8 (133.0) * e f	
Tea with sugar				0.804
v00	9.9 (53.3)	12.4 (64.0)	11.3 (59.1)	
v01	6.8 (46.9)	8.3 (49.7)	6.3 (42.5)	
▲	−16.5 (103.3)	−27.5 (114.7)	−41.7 (133.7)	
Infusions without sugar				0.296
v00	37.3 (104.6)	46.1 (139.5)	50.2 (121.4)	
v01	57.5 (123.4)	61.4 (129.7)	54.1 (116.8)	
▲	20.2 (141.2) *	15.3 (152.4)	3.9 (148.4)	
Infusions with sugar				0.576
v00	15.4 (70.2)	21.1 (81.1)	15.4 (74.6)	
v01	10.8 (56.6)	16.1 (70.6)	16.8 (73.5)	
▲	−4.6 (89.1)	−5.1 (94.9)	1.4 (96.4)	
Beer with and without alcohol				<0.001
v00	68.3 (156.6) b	118.5 (275.1) c	194.1 (369.2) b c	
v01	141.9 (304.4) a b	95.9 (275.6) a	57.8 (144.2) b	
▲	73.5 (301.0) * d e	−22.5 (287.1) d f	−136.2 (363.6) * e f	
Wine				<0.001
v00	44.6 (84.8) a b	64.9 (109.9) a	82.6 (123.3) b	
v01	61.2 (96.7)	69.5 (110.1)	65.6 (99.2)	
▲	16.5 (90.4) * e	4.6 (109.8) f	−17.1 (119.2) * e f	
High proof drinks				0.908
v00	2.1 (10.9)	2.7 (16.1)	2.6 (12.2)	
v01	2.0 (9.7)	1.3 (8.3)	1.4 (8.0)	
▲	−0.1 (13.1)	−1.3 (17.5)	−1.2 (14.3)	
Combined drinks				0.075
v00	1.2 (9.0)	3.7 (31.7)	4.5 (30.7)	
v01	2.7 (22.8)	3.4 (31.7)	1.4 (7.7)	
▲	1.5 (24.5) e	−0.3 (39.5)	−3.1 (30.6) * e	
Energy drinks				0.645
v00	0.1 (2.2)	1.1 (21.1)	0.4 (8.7)	
v01	0.0 (0.0)	0.2 (5.2)	0.0 (0.0)	
▲	−0.1 (2.2)	−0.8 (15.8)	−0.4 (8.7)	
Isotonic drinks				0.091
v00	1.4 (21.21)	0.7 (7.7)	3.1 (29.9)	
v01	1.2 (11.4)	6.5 (85.6)	0.6 (8.9)	
▲	−0.2 (24.1)	5.7 (86.1) *	−2.5 (30.1)	
Replacement shakes				0.223
v00	0.0 (0.0)	0.3 (5.7)	0.0 (0.0)	
v01	1.0 (20.6)	0.0 (0.0)	0.0 (0.0)	
▲	1.0 (20.6)	−0.3 (5.7)	0.0 (0.0)	

Values are mean (SD). Abbreviations: T1: Tertile 1. T2: Tertile 2. T3: Tertile 3. SD: Standard deviation. V00: Baseline; v01: one-year follow-up. ▲: Differences in beverage consumption between v00 and v01. ^§^ Differences by ANOVA in the beverage sustainability score between baseline and at 1-year follow-up distributed in tertiles: T1: beverage score reduction: −4 through −1; T2: no score changes; T3: beverage: 1 through 4. * v00 vs. v01. Different letters mean Bonferroni’s post hoc test: ^a^ T1 v00 vs. T2 v00; ^b^ T1 v00 vs. T3 v00; ^c^ T2 v00 vs. T3 v00; ^d^ T1 v01 vs. T3 v01; ^e^ T1 v01 vs. T3 v01; ^f^ Ts v01 vs. T3 v01. Greenhouse gas emissions, water use, energy use, and land use were considered for the sustainability score calculations. General linear model was calculated between baseline and year 1 and was adjusted by total energy intake and intervention group.

## Data Availability

There are restrictions on the availability of data for the PREDIMED-Plus trial, due to the signed consent agreements around data sharing. Requestors wishing to access the PREDIMED-Plus trial data used in this study can make a request to the PREDIMED-Plus trial Steering Committee chair: jordi.salas@urv.cat. The request will then be passed to members of the PREDIMED-Plus Steering Committee for deliberation.

## Abbreviations

BMI: body mass index; CO_2_eq: carbon dioxide equivalent; FFQ: food frequency questionnaire; GHGs: greenhouse gas emissions; MedDiet: Mediterranean diet; MetS: metabolic syndrome; PB: plant based; SD: standard deviation; Pt: echo indicator point; T1: tertile 1; T2: tertile 2; T3: tertile 3; v00: baseline; v01: one-year follow-up; GLM: general linear model; METs: metabolic equivalents of task.
